# Effect of Carbohydrate Content in a Pre-event Meal on Endurance Performance-Determining Factors: A Randomized Controlled Crossover-Trial

**DOI:** 10.3389/fspor.2021.664270

**Published:** 2021-05-28

**Authors:** Mats Holst Aandahl, Dionne A. Noordhof, Arnt Erik Tjønna, Øyvind Sandbakk

**Affiliations:** ^1^Department of Circulation and Medical Imaging, Faculty of Medicine and Health Sciences, Norwegian University of Science and Technology, Trondheim, Norway; ^2^Department of Neuromedicine and Movement Science, Centre for Elite Sports Research, Norwegian University of Science and Technology, Trondheim, Norway; ^3^Central Administration, St. Olavs Hospital, NeXt Move Core Facility, The University Hospital, Trondheim, Norway

**Keywords:** energy expenditure, lactate threshold, maximal oxygen uptake, oxygen cost, work economy, running economy, sports nutrition, time to exhaustion

## Abstract

The current study aimed to investigate the effect of the relative CHO content in a pre-event meal on time to exhaustion (TTE), peak oxygen uptake ($\overset{∙}{\text{V}}{\text{O}}_{2\text{peak}}$), the 2nd lactate threshold (LT2), onset of blood lactate accumulation (OBLA), and work economy (WE) and to compare responses between well-trained and recreationally trained individuals. Eleven well-trained and 10 recreationally trained men performed three trials in a randomized cross-over design, in which they performed exercise tests (1) after a high-CHO pre-event meal (3 g · kg^−1^), (2) a low-CHO pre-event meal (0.5 g · kg^−1^), or (3) in a fasted-state. The test protocol consisted of five submaximal 5-min constant-velocity bouts of increasing intensity and a graded exercise test (GXT) to measure TTE. A repeated measure ANOVA with a between-subjects factor (well-trained vs. recreational) was performed. A main effect of pre-event meal was found (*p* = 0.001), with TTE being 8.0% longer following the high-CHO meal compared to the fasted state (*p* = 0.009) and 7.2% longer compared to the low-CHO meal (*p* = 0.010). No significant effect of pre-event meal on $\overset{∙}{\text{V}}{\text{O}}_{2\text{peak}}$, LT2, OBLA, or WE (*p* ≥ 0.087) was found and no significant interaction effect between training status and pre-event CHO intake was found for TTE or any of the performance-determining variables (*p* ≥ 0.257). In conclusion, high-CHO content in the pre-event meal led to a longer TTE compared to a meal with a low-CHO content or exercising in a fasted state, both in well-trained and recreationally trained participants. However, the underlying physiological reason for the increased TTE is unclear, as no effect of pre-event meal on the main physiological performance-determining variables was found. Thus, pre-event CHO intake should be standardized when the goal is to assess endurance performance but seems to be of less importance when assessing the main performance-determining variables.

## Introduction

Carbohydrate (CHO) is a key fuel source during high-intensity exercise (Åstrand and Rodahl, 1977; Romijn et al., 1993; Hawley and Leckey, 2015) and pre-event exogenous CHO ingestion has been proved to be beneficial for endurance capacity and performance (Wright et al., 1991; Chryssanthopoulos et al., 2002; Davison et al., 2008; Tokmakidis and Karamanolis, 2008; Thomas et al., 2016). The potential mechanisms underlying improved endurance performance from pre-event CHO ingestion are linked to increased CHO availability, primarily liver glycogen after an overnight sleep, but also possibly a small increase in muscle glycogen (Nilsson and Hultman, 1973; Chryssanthopoulos and Williams, 1997). In turn, this increase in availability increases CHO oxidation during exercise (Wright et al., 1991; Chryssanthopoulos et al., 2002). For pre-exercise CHO ingestion to induce optimal effects on endurance performance it is important that the pre-exercise CHO meal is timed properly and is of sufficient size to account for the shift in substrate utilization (i.e., fat to CHO) and the increase in insulin levels, resulting in a decline in blood glucose levels (Sherman et al., 1991; Wright et al., 1991; Marmy-Conus et al., 1996; Burke et al., 2011). For pre-event fueling, it is recommended to consume 1–4 g per kg body mass in the 1–4 h before exercise (Thomas et al., 2016). While the effect of the pre-exercise CHO on endurance performance is rather clear, the effect of pre-exercise CHO on performance-determining variables remains less clear.

The three most important performance-determining factors explaining endurance performance are maximal oxygen uptake ($\overset{∙}{\text{V}}{\text{O}}_{2\text{max}}$), 2nd lactate threshold (LT2), and work economy (WE) (Pate and Kriska, 1984; Helgerud, 1994; Joyner and Coyle, 2008). An effect of pre-exercise CHO ingestion on one or more of the three mentioned performance-determining variables might contribute to the CHO-induced improvements in endurance performance. $\overset{∙}{\text{V}}{\text{O}}_{2\text{max}}$ reflects the body's ability to supply and utilize O_2_ at maximal exercise intensity and is a strong predictor of aerobic endurance performance (Saltin and Astrand, 1967; di Prampero et al., 1986). $\overset{∙}{\text{V}}{\text{O}}_{2\text{max}}$ appears to be limited by the supply of oxygen to the working muscles (Wagner, 1996; Bassett and Howley, 2000) in which exogenous CHO intake provides a minor influence (Yoshida, 1984; Mikulski et al., 2008). Therefore, it is unlikely that pre-exercise CHO ingestion has a measurable effect on $\overset{∙}{\text{V}}{\text{O}}_{2\text{max}}$ (Yoshida, 1984; Mikulski et al., 2008).

Lactate threshold has been defined as the second rise in blood lactate concentration ([La^−^]b) above baseline (Joyner and Coyle, 2008; Faude et al., 2009). Onset of blood lactate accumulation (OBLA) represents the point where [La^−^]b reaches 4 mmol · L^−1^ and is regarded a surrogate measurement for maximal lactate steady state (Heck et al., 1985; Faude et al., 2009). Although this concept is highly controversial, these thresholds are used to indicate the relative intensity that can be sustained for a prolonged period of time (Åstrand and Rodahl, 1977; Pollock, 1977). As glycogen is the primary substrate for [La^−^]b production, the state of the muscle glycogen stores has a clear influence on [La^−^]b (Hughes et al., 1982; Heigenhauser et al., 1983; Podolin et al., 1991; Sabapathy et al., 2006). However, the effect of the CHO content of the pre-event meal seems to be unclear. Currently, there are studies demonstrating lower LT2 and OBLA after a high CHO pre-event diet (Yoshida, 1984; Busse et al., 1991; Langfort et al., 2001) but the same effect has not been shown by studies in which CHO was ingested immediately before exercise (Ivy et al., 1981; Rotstein et al., 2007). There is currently a lack of studies investigating the effect of the CHO content of the pre-event meal in line with current sports nutrition recommendations (Thomas et al., 2016) that can alter both substrate utilization and muscle glycogen stores (Chryssanthopoulos et al., 2002).

In the current study, WE is referred to as the energy cost (EC) of running, as oxygen cost (OC) is to a much larger extent influenced by substrate utilization (Jeukendrup and Wallis, 2005; Rapoport, 2010; Shaw et al., 2013; Burke et al., 2017). The few studies that have investigated the effect of CHO ingested before and during running exercise on WE have not found a significant effect (Sproule, 1998; Brisswalter et al., 2000). Contrary, Dumke et al. (2007) found that CHO ingestion before and during exercise led to an improved cycling efficiency and economy. In addition, Cole et al. (2014) demonstrated that a three-day high CHO diet led to improved cycling efficiency compared to both a moderate and a low CHO diet. Later the same group found that CHO ingested during exercise attenuated the exercise-induced reduction in gross efficiency during exercise (Cole et al., 2018). Although several studies looked at the effect of exogenous CHO on efficiency or WE, none of the studies specifically looked at only the effect of the CHO content of the pre-event meal on efficiency or economy.

There are indications that exogenous CHO oxidation is higher during exercise in endurance-trained individuals compared to untrained individuals (Burelle et al., 1999), which might result in sparing of liver glycogen in trained athletes. The higher CHO oxidation can be explained by trained individuals' larger non-insulin-mediated glucose transport in the muscles and higher insulin sensitivity and thus, greater CHO uptake (King et al., 1988; Burelle et al., 1999; Greiwe et al., 1999; Bowden and McMurray, 2000). These differences in CHO uptake and utilization between trained and untrained individuals (Jansson and Kaijser, 1987; Burelle et al., 1999) might affect the effect of the CHO content of the pre-event meal on endurance performance and performance-determining variables. However, the response to exogenous pre-event CHO in a real-life meal has not sufficiently been compared between endurance-trained individuals and untrained individuals in relation to endurance performance. Such information has important implications for the standardization of CHO intake in future testing practice both in research and sports practice.

The primary aim of the current study was to investigate the effect of the relative CHO content in a pre-event meal on time to exhaustion (TTE) assessed during a graded exercise test (GXT), peak oxygen uptake ($\overset{∙}{\text{V}}{\text{O}}_{2\text{peak}}$), LT2, OBLA, and WE, and to compare these responses between well-trained endurance athletes and recreationally trained individuals. We expected that a high-CHO content in the pre-event meal would positively influence the TTE but would not affect LT2 or $\overset{∙}{\text{V}}{\text{O}}_{2\text{peak}}$. In addition, we hypothesized that a pre-event meal with a high CHO content would have a stronger effect on TTE and [La^−^]b in the well-trained participants compared to the recreationally trained.

## Materials and Methods

### Participants

Twenty-one healthy, non-smoking men participated in this study. Participants' characteristics can be found in Table 1. The participants were divided into a well-trained (*N* = 11) and recreationally trained (*N* = 10) group. The well-trained participants comprised competitive rowers, runners, and triathletes who were non-professional but still training once or twice each day. All participants had to review and sign a consent form and go through a health-history questionnaire before being allowed to participate in the study. In the recreationally trained group, a $\overset{∙}{\text{V}}{\text{O}}_{2\text{max}}$ of 53 ml · kg^−1^ · min^−1^ was set as the upper limit for the participants' aerobic capacity, in order to be below the average $\overset{∙}{\text{V}}{\text{O}}_{2\text{max}}$ in Norwegian men aged 21–29 years old (De Pauw et al., 2013; Loe et al., 2014). The well-trained participants needed to have a $\overset{∙}{\text{V}}{\text{O}}_{2\text{max}}$ of at least 60 ml · kg^−1^ · min^−1^ (De Pauw et al., 2013).

**Table 1:** Participant characteristics.

	**Well-trained**	**Recreationally trained**
	**(*n* = 11)**	**(*n* = 10)**
Age (years)	24.6 (2.3)	25.1 (3.5)
Height (cm)	183.1 (5.9)	179.4 (6.5)
BM (kg)	77.8 (9.2)^*^	92.3 (12.6)
BMI (kg · m^2^)	23.2 (1.9)^*^	28.7 (4.4)
$\overset{∙}{\text{V}}{\text{O}}_{2\text{max}}$ (L · min^−1^)	5.6 (0.6)^**^	4.3 (0.7)
$\overset{∙}{\text{V}}{\text{O}}_{2\text{max}}$ (ml · kg^−1^ · min^−1^)	71.9 (5.1)^**^	46.9 (2.5)
HRmax (beats · min^−1^)	197.9 (6.9)	199.5 (5.0)

*Data are presented as mean (SD), BM, body mass measured at baseline; BMI, body mass index; V°O_2_, oxygen uptake; HR, heart rate.*

* *significant differences between groups (p ≤ 0.05),*

** *significant differences between groups (p <0.001)*.

### Ethics Statement

The Regional Committee for Medical and Health Research Ethics waives the requirement for ethical approval for such studies. Therefore, the ethics of the study is done according to the institutional requirements, and approval for data security and handling was obtained from the Norwegian Centre for Research Data. Prior to the data collection, participants were informed about the protocol and the possibility to withdraw from the study at any point without providing a reason, after which they provided written informed consent.

### Study Design

**Figure 1:**
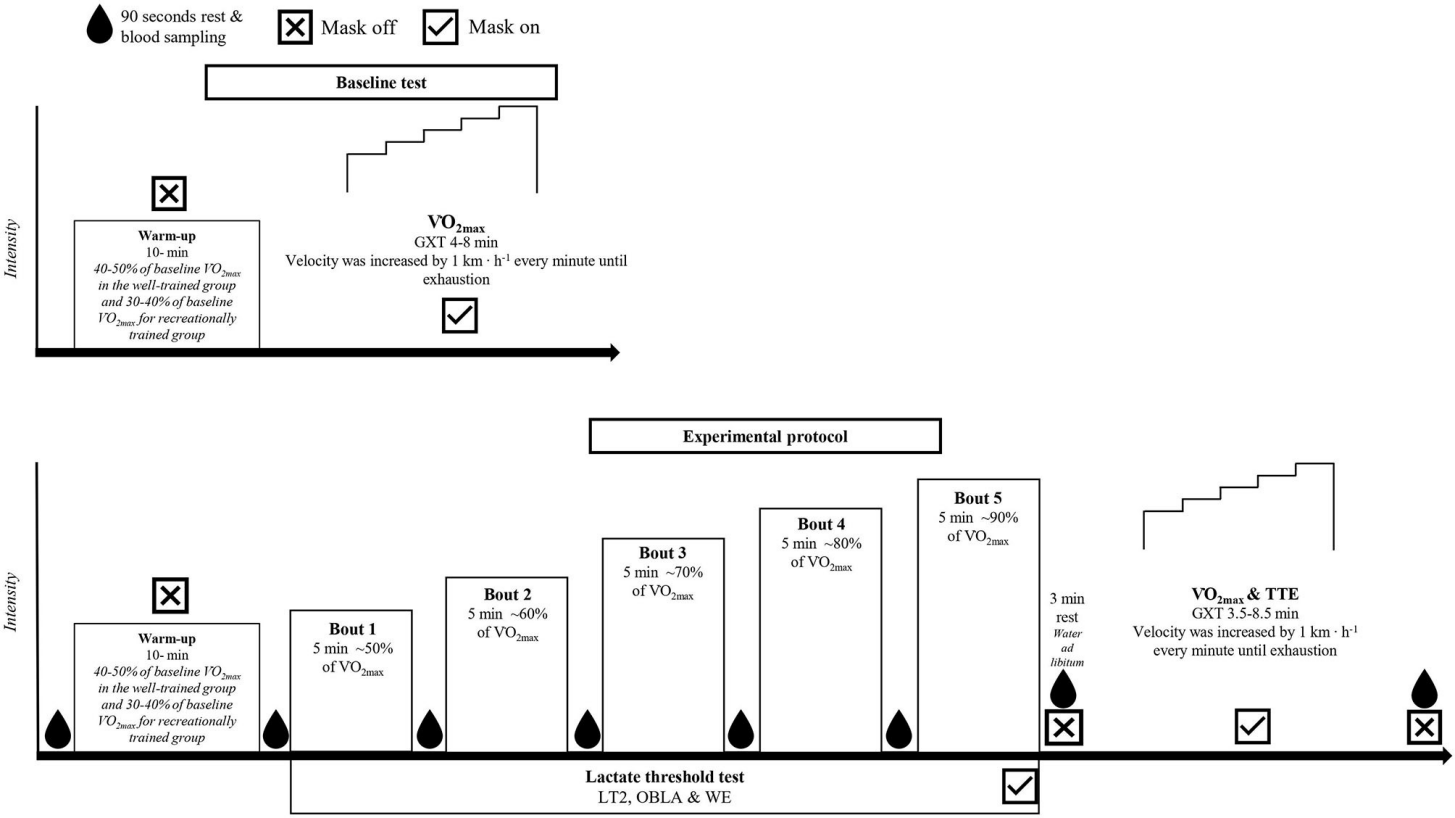
Study design illustrating the initial baseline test and the experimental protocol for the three trials. $\overset{∙}{\text{V}}{\text{O}}_{2\text{max}}$, maximal oxygen uptake measured at the baseline test; GXT, graded exercise test; LT2, lactate threshold; OBLA, onset of blood lactate accumulation; WE, work economy; TTE, time to exhaustion.

This study was a randomized controlled cross-over trial. Participants reported to the laboratory on 4 occasions. The tests included GXT for the determination of baseline $\overset{∙}{\text{V}}{\text{O}}_{2\text{max}}$ and familiarization (see Figure 1). Subsequently, three experimental trials in randomized order were conducted, all three trials were performed while running on a treadmill with an inclination of 5.3%. The trials were separated by a minimum of 6 days. During the GXT of both the initial baseline test and at the end of the experimental trials three trials, velocity was increased by 1 km · h^−1^ every minute until exhaustion.

#### Experimental Protocol

The protocol of the experimental trials consisted of a warm-up, LT test, and a GXT (Figure 1). The warm-up consisted of 10-min running aimed at 40–50% of baseline $\overset{∙}{\text{V}}{\text{O}}_{2\text{max}}$ for the well-trained group and 30–40% of $\overset{∙}{\text{V}}{\text{O}}_{2\text{max}}$ for the recreational group. The LT test consisted of 5 ×5-min exercise bouts with increasing intensities to establish LT2, OBLA, and determine WE (Faude et al., 2009; Barnes and Kilding, 2015). The velocity of the bouts was calculated to result in an intensity approximating 50, 60, 70, 80, and 90% of participants' baseline $\overset{∙}{\text{V}}{\text{O}}_{2\text{max}}$. There 155 was a 90-s break between the bouts for sampling of capillary blood from the fingertip to determine [La^−^]b and blood glucose (GLU). After a 3-min break following the last bout of the LT test, participants completed a GXT to establish TTE and $\overset{∙}{\text{V}}{\text{O}}_{2\text{peak}}$. The GXT started at 2 km · h^−1^ slower velocity than the highest velocity of the LT test and the subsequent velocity increased by 1 km · h^−1^ every minute until exhaustion. Respiratory and heart rate (HR) data were collected during the LT test and GXT. Capillary blood for [La-]b determination was sampled at rest before the warm-up, after the warm-up, after the GXT, and in-between each bout of the LT test. During the 3-min break between the LT test and the GXT, participants could drink water *ad libitum*. Prior to all test days, participants were told to refrain from any strenuous exercise 48 h prior to the tests and to refrain from all exercise 24 h prior.

### Nutritional Protocol

The three pre-event meal conditions for the experimental trials were as following: (1) a minimum of 12 h of fasting before the test. (2) A standardized pre-event meal with a high CHO content (3 g · kg^−1^ CHO), which consisted of white bread, jam, skimmed milk, oats, banana, and raisins and was based on the pre-event meal provided in Chryssanthopoulos et al. (2002) and in line with current recommendations (Thomas et al., 2016); (3) an isoenergetic pre-event meal with a low CHO content (0.5 g · kg^−1^ CHO) given to participants, which consisted of yogurt, almonds, and avocado. Both meals were given three and a half hours prior to the tests. During the 3 days prior to the three experimental trials, participants were told to record and replicate their diet to minimize differences in glycogen stores between the test days. Participants recorded their diets 3 days prior to each trial using the app “dietist.net,” which was later analyzed by a sports nutritionist. Lastly, participants were told to refrain from the use of caffeine in the last 24 h prior to all trials.

### Data Collection

Testing was performed on a treadmill (Woodway PPS 55, Waukesha, WI, USA). Respiratory data were obtained using an ergospirometry system with a mixing chamber (Metalyzer II, CORTEX Biophysik GmbH, Leipzig, Germany), with 10-s mixing chamber values being used for analyses. [La^−^]b was established using Biosen C-line lactate analyzer (EKF-diagnostic GmbH, Leipzig, Germany), and GLU using HemoCue Glucose 201 RT (HemoCue AB, Ängelholm, Sweden).

### Data Analysis

#### Maximal and Peak Oxygen Uptake

$\overset{∙}{\text{V}}{\text{O}}_{2\text{max}}$ of the baseline test and $\overset{∙}{\text{V}}{\text{O}}_{2\text{peak}}$ of the three experimental trials were determined from the highest average of three consecutive 10-s outputs from the GXT. V°O_2_ was accepted as $\overset{∙}{\text{V}}{\text{O}}_{2\text{max}}$ if the first and two of the remaining three criteria were met: (1) a plateau or a decrease in V°O_2_ despite an increase in workload, (2) a RER ≥ 1.05, (3) [La^−^]b > 7 mmol · l^−1^, and (4) a rate of perceived exertion (RPE) >18 on the Borg scale (Helgerud et al., 1990; Howley et al., 1995; Meyer et al., 2005; Midgley et al., 2007). During the experimental trials, the criteria of the plateau was met for less than half of the tests, for this reason, measurements from the three experimental trials are defined as $\overset{∙}{\text{V}}{\text{O}}_{2\text{peak}}$ and not $\overset{∙}{\text{V}}{\text{O}}_{2\text{max}}$ (Meyer et al., 2005; Midgley et al., 2007). Peak velocity (vLpeak) was determined from the highest velocity each participant reached during the GXT.

#### Lactate Threshold and Onset of Blood Lactate Accumulation

LT2 from the experimental trials is considered to be the intensity at which [La^−^]b was 2.3 mmol · l^−1^ above the individual warm-up [La^−^]b value (adapted from Helgerud et al., 1990) to be appropriate for hemolyzed blood samples (Helgerud et al., 1990; Medbo et al., 2000; Faude et al., 2009) and OBLA is considered to be the intensity corresponding to a [La^−^]b of 4 mmol · l^−1^. LT2 and OBLA, expressed as a % of V°O2peak, were determined by fitting an exponential function (a + b · exp(c · x)), start points [1 1.1 1] to the [La^−^]b vs. %V°O2peak data. Subsequently, the velocity corresponding to LT2 (vLT2) and OBLA (vOBLA) was determined by fitting the exponential equation to the [La^−^]b vs. velocity data. The highest heart rate measurement regardless of the test was used as HRmax and was assessed using a Polar H7 HR transmitter (Polar Electro, Kempele, Finland).

#### Work Economy

The average V°O_2_ and V°CO_2_ from the last minute of each of the five exercise bouts in the LT test were used to establish WE (EC). This was done using a non-protein respiratory quotient equation by Peronnet and Massicotte (1991). Since EC could only be validly calculated with this method if RER was <1.00, the highest exercise intensity where the average RER during the final minute was <1.00 was used to calculate EC for each participant. For intra-individual comparisons, the exercise bout used to compare the three different trials was the same bout (i.e., if RER exceeded 1.00 in the 3rd bout for a participant this bout was used to compare EC between experimental trials regardless of whether RER did not exceed 1.00 in the 3rd bout in the two remaining trials).

### Statistical Analysis

Data are presented as means (SDs). The assumption of normality was checked for each variable using quantile-quantile (QQ) plots, histogram, and Shapiro-Wilk test. In all cases, *P* ≤ 0.05 was used as the level of significance. To assess the effect of the pre-event and training status, a two-way mixed factor ANOVA was used. This was followed up by a one-way repeated measure ANOVA for each of the two groups separately if the interaction effect was significant, and with both groups combined if the main effect of pre-event meal was significant, but the interaction effect was not. When Mauchly's test for sphericity was violated for the two-way ANOVA, the Greenhouse- Geisser correction was used. Whenever the main effects of pre-event meals were significant, pair-wise comparisons were made using Bonferroni adjustments. The effect size using partial eta squared ($\eta_{\text{p}}^{2}$) was used to describe effects of group, pre-event meal and related interaction effects. The software program IBM SPSS, version 26.0 (Statistical Package for Social Science, Chicago, IL, USA) was used for the statistical analysis.

## Results

Average CHO intake for all three, three-day periods were 3.3 (0.8) g per kg body of mass for the well-trained group and 2.4 (0.8) g per kg body of mass for the recreationally trained group. No difference in mean CHO intake (_Well−trained_ [*F*_(2, 12)_ = 0.67, *p* = 0.532, η${}_{\text{p}}^{2}$ = 0.01]), (_Recreational_ [*F*_(2, 12)_ = 3.50, *p* = 0.064, η${}_{\text{p}}^{2}$ = 0.37]) or energy intake (_Well−trained_ [*F*_(2, 12)_ = 2.52, *p* = 0.122, η${}_{\text{p}}^{2}$ = 0.30]), (_Recreational_ [*F*_(2, 12)_ = 0.05, *p* = 0.948, η${}_{\text{p}}^{2}$ = 0.01]) was found between the 3 days prior to each of the three trials.

CHO content of the pre-event meal influenced TTE attained during the GXT [*F*_(2, 38)_ = 8.14, *p* = 0.001, η${}_{\text{p}}^{2}$ = 0.30] (Table 2). TTE was higher following the high-CHO meal compared to both the fasted trial (*p* = 0.009, Cl 95%: 6.49–49.31) and the low-CHO meal (*p* = 0.013, Cl 95%: 4.60–45.01). No interaction effect was found between the CHO content of the pre-event meal and training status [*F*_(2, 36)_ = 0.19, *p* = 0.828, η${}_{\text{p}}^{2}$ = 0.01]. There was no main effect of training status on TTE [*F*_(2, 36)_ = 0.06, *p* = 0.802, η${}_{\text{p}}^{2}$ = 0.00]. An effect of CHO content in the pre-event meal was found on vLpeak [*F*_(2, 38)_ = 7.51, *p* = 0.001, η${}_{\text{p}}^{2}$ = 0.29]. vLpeak was higher following the high-CHO meal compared to the fasted trial (*p* < 0.001, Cl 95%: 0.15–0.95) and the low-CHO meal (*p* = 0.02, Cl 95%: 0.05–0.94).

**Table 2:** Time to exhaustion and the peak velocity attained during the graded exercise test in well-trained and recreationally trained participants.

	**Well-trained (*****n*** **=** **11)**			**Recreationally trained (*****n*** **=** **10)**		
	**High CHO**	**Low CHO**	**Fasted**	**High CHO**	**Low CHO**	**Fasted**
TTEramya (min:ss.ss)	5:52.00 (1:31.45)^*^	5.30.30 (1:24.09)	5:22.50 (1:09.04)	5:45.21 (0:51.28)	5:17.30 (1:03.63)	5:18.90 (1:01.66)
vLpeakramya (km · h^−1^)	17.5 (1.5)^*^	16.9 (1.4)	16.9 (1.7)	11.3 (1.1)	10.8 (1.5)	10.9 (1.4)

*Data are presented as mean (SD). High CHO, pre-event meal with high carbohydrate content; low CHO, pre-event meal with low carbohydrate content; fasted, test in fasted state; TTE, Time to exhaustion from the respective trial stated as minute: seconds; vLpeak, peak velocity attained during the graded exercise test in the respective trial.*

* *significant effect of pre-event meals after a follow-up test with both groups included (p ≤ 0.05)*.

There was no effect of CHO content of the pre-event meal on $\overset{∙}{\text{V}}{\text{O}}_{2\text{peak}}$, LT2, OBLA, and WE (*p* > 0.087, η${}_{\text{p}}^{2}$ < 0.12). In addition, there was no interaction effect between CHO content of the pre-event meal and training status on these variables (*p* > 0.099) (Table 3). No main effect of training status was found for LT2 or OBLA (*p* > 0.100); however, WE was lower in the well-trained group compared to the recreationally trained group (*p* < 0.004).

**Table 3:** Effect of pre-event meal on peak oxygen uptake, lactate threshold, and work economy in well-trained and recreationally trained participants.

	**Well-trained (*****n*** **=** **11)**			**Recreationally trained (*****n*** **=** **10)**		
	**HIGH CHO**	**LOW CHO**	**FASTED**	**HIGH CHO**	**LOW CHO**	**FASTED**
$\overset{∙}{\text{V}}{\text{O}}_{2\text{peak}}$ramya L · min^−1^	5.21 (0.64)	5.15 (0.69)	5.21 0.67)	4.17 (0.69)	4.00 (0.63)	4.07 (0.70)
$\overset{∙}{\text{V}}{\text{O}}_{2\text{peak}}$ramya ml · kg^−1^ · min^−1^	68.0 (7.4)	67.1 (7.4)	67.9 (5.3)	45.1 (2.5)	43.4 (2.8)	44.3 (2.6)
LT2 % $\overset{∙}{\text{V}}{\text{O}}_{2\text{max}}$	73.6 (5.8)	73.3 (7.2)	73.5 (3.9)	67.2 (8.4)	69.3 (9.4)	67.0 (10.0)
OBLA % $\overset{∙}{\text{V}}{\text{O}}_{2\text{max}}$	75.0 (5.5)	76.1 (6.5)	76.8 (4.6)	70.9 (8.7)	73.0 (7.8)	70.3 (8.5)
_v_LT2 (km · h^−1^)	12.4 (1.5)	12.2 (1.5)	12.1 (1.6)	6.5 (1.2)	6.5 (1.1)	6.3 (1.2)
_v_OBLA (km · h^−1^)	12.8 (1.5)	12.6 (1.5)	12.6 (1.5)	6.7 (1.1)	6.8 (1.1)	6.6 (1.2)
[La^−^]b	3.7 (0.4)^*^	3.3 (0.2)	3.4 (0.3)	3.7 (0.4)	3.4 (0.5)	3.5 (0.5)
WE Absoluteramya (Kcal · km^−1^)	101.7 (16.0)	103.4 (12.2)	104.3 (17.0)	142.5 (22.9)	149.5 (20.7)	146.6 (24.0)
WE SI-unitsramya (J · kg^−1^ · m^−1^)	5.5 (0.6)	5.6 (0.6)	5.6 (0.5)	6.5 (0.9)	6.9 (1.3)	6.7 (1.1)
Oxygen costramya (l · min^−1^)	3.86 (0.54)^*^	4.06 (0.62)	4.06 (0.55)	2.96 (0.76)^*^	3.05 (0.77)	3.05 (0.71)
Body mass (kg)	77.5 (8.7)	77.5 (9.2)	77.5 (9.0)	92.1 (11.3)	91.7 (12.1)	92.1 (12.5)

*Data are presented as mean (SD). V°O_2_, oxygen uptake; LT2, second lactate threshold; OBLA, onset of blood lactate accumulation; _v_LT2 and _v_OBLA, velocity at lactate threshold and onset of blood lactate accumulation; [La^−^]b, individual lactate concentration after warm-up + 2.3; WE, highest accepted energy cost value calculated from the last bout where RER was < 1.00; Oxygen cost, oxygen cost value equivalent to the energy cost value, body mass in kilograms; High CHO, pre-event meal with high carbohydrate content; low CHO, pre-event meal with low carbohydrate content; fasted, test in fasted state,*

* *significant main effect of pre-event meal (p ≤ 0.05)*.

**Figure 2:**
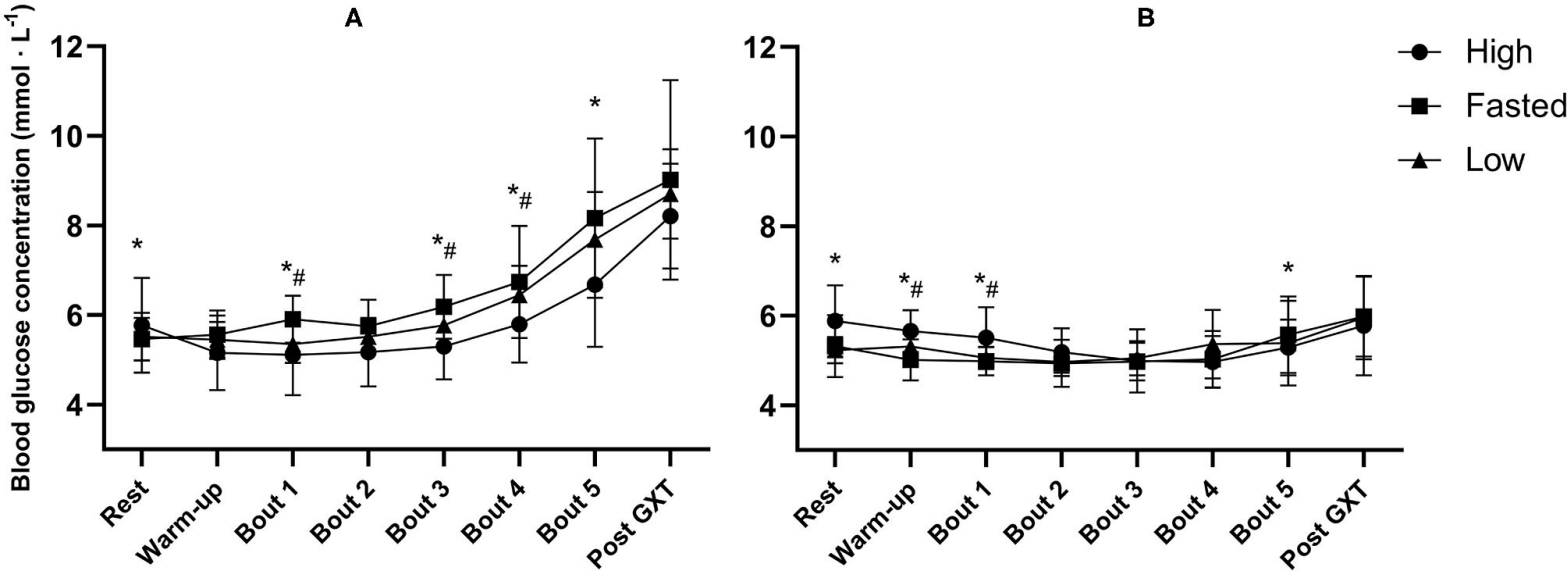
Mean blood glucose concentrations before and after warm-up, following all five 5-min exercise bouts at ~50, 60, 70, 80, and 90% of $\overset{∙}{\text{V}}{\text{O}}_{2\text{max}}$, and after the graded exercise test (GXT) in well-trained **(A)** and recreationally trained **(B)**. High, pre-event meal with high carbohydrate content; low, pre-event meal with low carbohydrate content; fasted, test in fasted state. *significant effect of pre-event meals after a follow-up test with both groups included (*p* ≤ 0.05), *#significant effect of pre-event meals after a follow-up test for the two groups separately (*p* ≤ 0.05).

CHO content of the pre-event meal had an effect on mean GLU in the well-trained group [*F*_(2, 20)_ = 4.89, *p* = 0.019, η${}_{\text{p}}^{2}$ = 0.33] but not in the recreationally trained group [*F*_(2, 18)_ = 0.72, *p* = 0.718, η${}_{\text{p}}^{2}$ = 0.07] (Figure 2). An interaction effect between training status and the effect of the pre-event meal was found for mean GLU [*F*_(2, 38)_ = 5.13, *p* = 0.011, η${}_{\text{p}}^{2}$ = 0.21]. In the well-trained group mean GLU was 0.73 mmol · L^−1^ lower following the high-CHO trial compared to the fasted-trial (*p* = 0.042, Cl 95%: −1.44 to −0.02) and 0.43 mmol · L^−1^ lower compared to the low-CHO trial (*p* = 0.383, Cl 95%: −1.17–0.31). There was a main effect of training status on GLU, mean GLU was higher in the well-trained group compared to the recreationally trained group [*F*_(1, 19)_ = 26.49, *p* < 0.001, η${}_{\text{p}}^{2}$ = 0.58].

**Figure 3:**
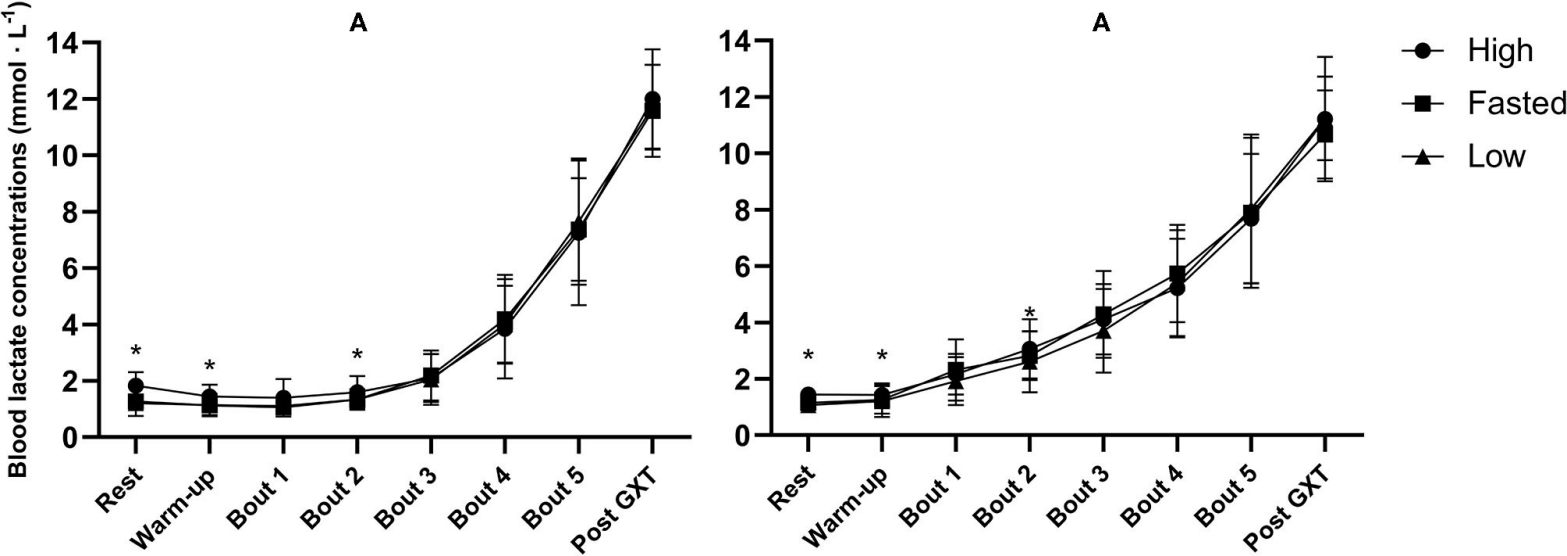
Mean blood lactate concentrations before and after warm-up, following all five 5-min exercise bouts at ~50, 60, 70, 80, and 90% of $\overset{∙}{\text{V}}{\text{O}}_{2\text{max}}$, and after the graded exercise test (GXT) in well-trained **(A)** and recreationally trained **(B)**. High, pre-event meal with high carbohydrate content; low, pre-event meal with low carbohydrate content; fasted, test in fasted state. *significant effect of pre-event meals after a follow-up test with both groups included (*p* ≤ 0.05).

No effect of CHO content of the pre-event meal was found on mean [La^−^]b [*F*_(2, 38)_ = 1.16, *p* = 0.326, η${}_{\text{p}}^{2}$ = 0.06] (Figure 3). Additionally, there was no interaction effect between training status and the effect of the pre-event meal [*F*_(2, 38)_ = 0.56, *p* = 0.579, η${}_{\text{p}}^{2}$ = 0.03]. A main effect of training status on mean [La^−^]b was found, [La^−^]b was higher in the recreationally trained participants for the first three exercise bouts compared to the well-trained participants {1st: [*F*_(1, 19)_ = 12.43, *p* = 0.002, η${}_{\text{p}}^{2}$ = 0.40]}, {2nd: [*F*_(1, 17)_ = 18.16, *p* <0.001, η${}_{\text{p}}^{2}$ = 0.52]}, {3rd: [*F*_(1, 19)_ = 15.74, *p* <0.001, η${}_{\text{p}}^{2}$ = 0.45]}.

**Figure 4:**
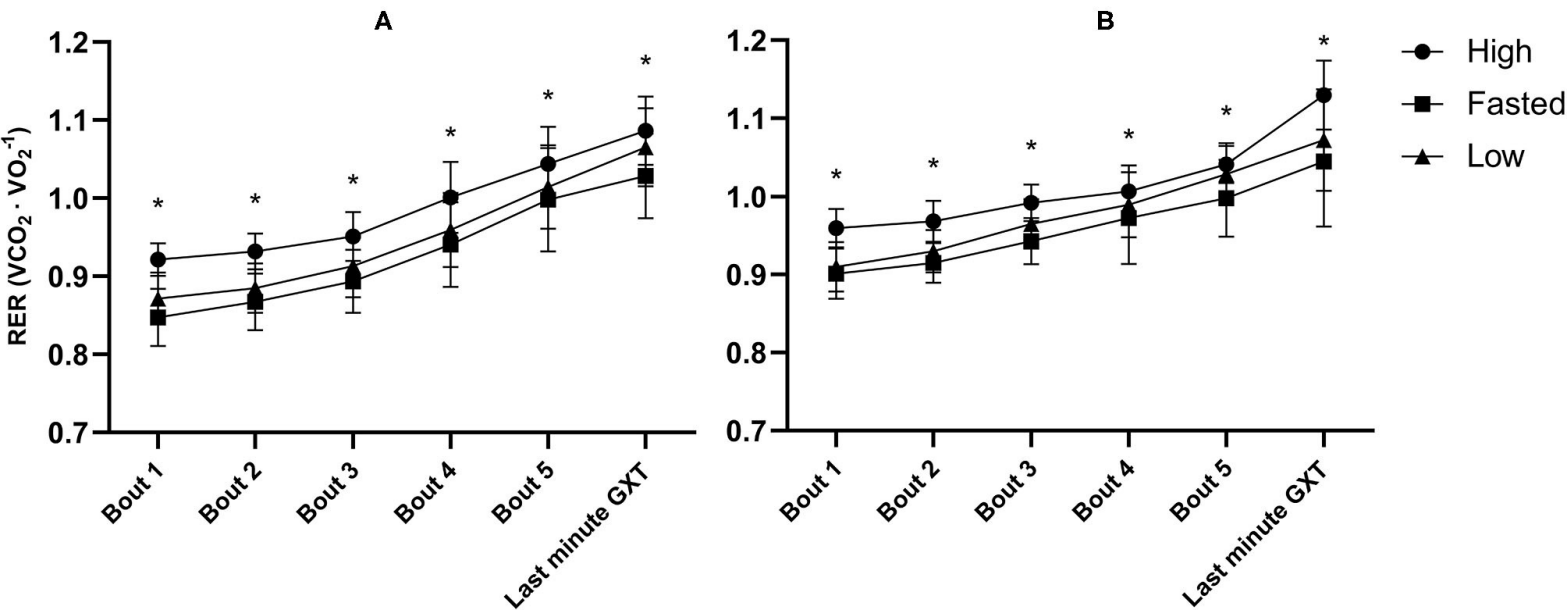
Mean respiratory exchange ratio (RER) during the final minute of all five exercise bouts at ~50, 60, 70, 80, and 90% of $\overset{∙}{\text{V}}{\text{O}}_{2\text{max}}$ and the graded exercise test (GXT) in well-trained **(A)** and recreationally trained **(B)**. High, pre-event meal with high carbohydrate content; low, pre-event meal with low carbohydrate content; fasted, test in fasted state. *significant effect of pre-event meals after a follow-up test with both groups included (*p* ≤ 0.05).

There was an effect of the pre-event meals on mean RER [*F*_(2, 40)_ = 31.81, *p* <0.001, η${}_{\text{p}}^{2}$ = 0.61] (Figure 4). Mean RER was 0.05 higher following the high-CHO trial compared to the fasted-trial (*p* <0.001, Cl 95%: 0.03–0.08) and 0.04 higher compared to low-CHO trial (*p* <0.001, Cl 95%: 0.02–0.05). Additionally, mean RER was higher following the low-CHO trial compared to the fasted trial (*p* = 0.048, Cl 95%: −0.04 to −0.00). There was a main effect of training status, RER was higher in the recreationally trained group for the first three exercise bouts compared to the well-trained group [*F*_(1, 17)_ > 15.26, *p* <0.001, η${}_{\text{p}}^{2}$ > 0.45].

**Figure 5:**
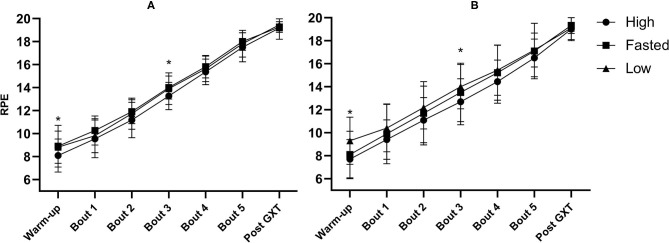
Mean rate of perceived exertion (RPE) after warm-up, following all five exercise bouts at ~50, 60, 70, 80, and 90% of $\overset{∙}{\text{V}}{\text{O}}_{2\text{max}}$ and after the graded exercise test (GXT) in well-trained **(A)** and recreationally trained **(B)**. High, pre-event meal with high carbohydrate content; low, pre-event meal with low carbohydrate content; fasted, test in fasted state. *significant effect of pre-event meals after a follow-up test with both groups included (*p* ≤ 0.05).

The CHO content of the pre-event meal had an effect on mean RPE [*F*_(2, 40)_ = 6.32, *p* = 0.004, η${}_{\text{p}}^{2}$ = 0.24] (Figure 5). Mean RPE was 0.63 lower following the high-CHO trial compared to the fasted trial (*p* = 0.084, Cl 95%: −1.33–0.07) and 0.77 lower compared to the low-CHO trial (*p* = 0.002, Cl 95%: −1.28 to −0.27).

## Discussion

The present study investigated the effect of the relative CHO content in a pre-event meal on TTE attained during the GXT, $\overset{∙}{\text{V}}{\text{O}}_{2\text{peak}}$, LT2, OBLA, and WE and compared the responses to pre-event CHO intake between well-trained and recreationally trained participants. The main finding was that TTE was 8.0% longer following the high-CHO meal compared to the fasted-state and 7.2% longer compared to the low-CHO meal with no significant effect of training status on these responses. However, no significant effect of the CHO content of the pre-event meal was found on $\overset{∙}{\text{V}}{\text{O}}_{2\text{peak}}$, LT2, OBLA, and WE. There were no differences in the responses to pre-event CHO between well-trained and recreationally trained participants for the different performance-determining variables. However, there were differences in how RER and GLU changed in response to increasing intensities between the two groups. Mean GLU was higher in the well-trained group compared to the recreationally trained group while mean RER was higher in the recreationally trained group.

TTE as a performance indicator has commonly been used to examine the influence of an experimental intervention on endurance performance both in lab and field testing (Hopkins et al., 2001; Amann et al., 2008). In the well-trained group, TTE was 8.5% longer following the high-CHO trial compared to the fasted trial and 6.2% longer compared to the low-CHO trial. In the recreationally trained group, TTE was 7.6% longer following the high-CHO trial compared to the fasted trial and 8.1% longer compared to the low-CHO trial (Table 2). Despite the relatively short duration of the current study's protocol (5–6 min of GXT following 35 min of incremental exercise), the percentage improvement of TTE is comparable with what other studies have reported using and different administration strategies and testing protocols (Chryssanthopoulos et al., 2002; Davison et al., 2008; Tokmakidis and Karamanolis, 2008). Thus, based on our findings, TTE measured during a GXT is influenced by relatively acute changes of pre-event CHO availability, regardless of the participants' training status.

### Performance-Determining Variables

No significant effect of the relative CHO content of pre-event meal on $\overset{∙}{\text{V}}{\text{O}}_{2\text{peak}}$ was found in neither the well-trained nor the recreationally trained participants, indicating that $\overset{∙}{\text{V}}{\text{O}}_{2\text{peak}}$ did not contribute to the improved TTE during the GXT and that the participants reached a similar $\overset{∙}{\text{V}}{\text{O}}_{2\text{peak}}$ at a faster rate and at a lower velocity following the fasted- and the low-CHO trial compared to the high-CHO trial. Our findings agree with the findings of Mikulski et al. (2008), who also found that the CHO content in a pre-event meal did not have a significant effect on $\overset{∙}{\text{V}}{\text{O}}_{2\text{peak}}$. Similar findings have also been found when using more impactful interventions such as 3–4 days dietary interventions and glycogen depletion protocols (Yoshida, 1984; Sabapathy et al., 2006). This is not surprising as $\overset{∙}{\text{V}}{\text{O}}_{2\text{peak}}$ is mainly dependent on oxygen supply to the working muscle (Wagner, 1996; Bassett and Howley, 2000).

There was no effect of the relative CHO content of pre-event meal on LT2, OBLA, vLT2, or vOBLA in either group, indicating neither of these variables contributed to the difference observed in TTE. This is in line with what Rotstein et al. (2007) and Ivy et al. (1981) reported when giving CHO immediately before and during exercise and with what Quirion et al. (1988) reported using two-day dietary interventions but in opposition to what Yoshida (1984) reported using 3–4 day dietary interventions. Yoshida (1984) found that a three-day high CHO diet led to OBLA occurring at a significantly lower percentage of $\overset{∙}{\text{V}}{\text{O}}_{2\text{max}}$ (66.3% of $\overset{∙}{\text{V}}{\text{O}}_{2\text{max}}$) compared to a three-day mixed diet (72.7% of $\overset{∙}{\text{V}}{\text{O}}_{2\text{max}}$) and a four-day low CHO diet (75.8% of $\overset{∙}{\text{V}}{\text{O}}_{2\text{max}}$) when measured during an incremental cycling exercise. Thus, OBLA does not seem to be influenced by acute changes in pre-exercise CHO availability to the same extent as by longer dietary interventions.

The effect of the pre-event meal on [La^−^]b was proposed to be dependent on the alteration of muscle glycogen stores and substrate utilization during exercise. Muscle glycogen was not measured but the timing and dosage of the high CHO meal should have been sufficient to increase the stores. Chryssanthopoulos et al. (2004), found an 11% increase in muscle glycogen of the m. vastus lateralis when using a highly comparable pre-test meal and timing. It can be speculated that a pre-event meal cannot sufficiently alter muscle glycogen and metabolism for there to be a measurable effect on LT2 and OBLA, which could explain the discrepancy between our findings and that of Yoshida (1984) and what was indicated in Busse et al. (1991). Another possible explanation for the lack of effect could be that glycogen stores only affect OBLA or LT2 when elevated stores are compared with depleted stores. Overall, our findings and others demonstrate that neither LT2 nor OBLA are influenced by acute changes in CHO availability induced by a pre-event meal (Ivy et al., 1981; Rotstein et al., 2007).

There was no effect of the relative CHO content of the pre-event meal on WE measured at 70–74% of $\overset{∙}{\text{V}}{\text{O}}_{2\text{max}}$ in the well-trained group and 68–70% of $\overset{∙}{\text{V}}{\text{O}}_{2\text{max}}$ in the recreationally trained group, indicating that WE did not contribute to the differences in TTE between trials. In line with our findings, Brisswalter et al. (2000) did not find a significant effect of 0.44 g · kg^−1^, from a 5.5% CHO solution immediately before exercise and 0.11 g · kg^−1^ every 20 min on EC determined after 3 min and 120 min of running at ~80% $\overset{∙}{\text{V}}{\text{O}}_{2\text{max}}$ in well-trained triathletes. Contrary, Dumke et al. (2007) found that 0.72 g · kg^−1^ of a 6% CHO solution given immediately before and 0.24 g · kg^−1^ · 15 min^−1^ during exercise led to significantly greater cycling efficiency after 40 min of cycling at ~75% $\overset{∙}{\text{V}}{\text{O}}_{2\text{max}}$ compared to the placebo trial. The discrepancy between the findings of Dumke et al. (2007) and those of the current study and Brisswalter et al. (2000) are possible due to differences in requirements between running and cycling and possibly the size of the CHO intake both before and during exercise which in both was largest in Dumke et al. (2007). Based on our findings it does not seem that alteration of the CHO content of pre-event meals significantly affects WE measured during treadmill running. Future studies should consider measuring the performance-determining variables after a prolonged period of 60 to 90 min of submaximal work to gain more valid insight into long-distance performance (Dumke et al., 2007; Noordhof et al., 2020) without the addition of CHO ingestion during exercise. Although no effect of CHO content of the pre-event meal was found on WE, we found an effect of pre-event meal on RER in both groups. Additionally, RER and GLU levels were to be directly related to the relative intensity of exercise in the well-trained participants. This occurs due to increasing intensity resulting in increasing GLU levels to cover the shift in metabolic requirements (Friedlander et al., 1997; Ramos-Jiménez et al., 2008). However, the increase in GLU levels in relation to exercise intensity was not seen in the recreationally trained participants where only RER increased with increasing intensity.

Since the effects on TTE could not be explained by the main performance-determining physiological variables, $\overset{∙}{\text{V}}{\text{O}}_{2\text{peak}}$, LT2, OBLA, and WE, other factors may have played a role. TTE was measured at relatively high intensity over a short duration, in which CHO will be the dominant fuel source (Romijn et al., 1993), and the higher rates of CHO oxidation following the high-CHO trial could have led to an improved anaerobic capacity (Lee et al., 2011; Michalczyk et al., 2019) and therefore allowed participants to sustain a higher running pace for longer time, resulting in longer TTE compared to the low-CHO and fasted trial. However, this is speculative as the effect of pre-exercise CHO on anaerobic performance was not assessed in the current study. Other factors at play could be beneficial effects of CHO on the central nervous system, by delaying central fatigue (Carter et al., 2004; Clark et al., 2019). Lastly, other effects of CHO solely related to delaying fatigue by attenuating the accumulation of ATP breakdown products without interacting with the performance-determining variables (Costill and Hargreaves, 1992; Clark et al., 2019). These fatigue delaying mechanisms should be further examined following real-life pre-event meals in future studies with the multifactorial nature of fatigue taken into consideration.

### Effect of Training Status

There was no significant difference in the responses to the relative CHO content of the pre-event meal on TTE between the well-trained and recreationally trained participants. Similarly, no significant differences in the responses to the pre-event meal on the performance-determining variables $\overset{∙}{\text{V}}{\text{O}}_{2\text{peak}}$, LT2, OBLA, and WE were found between the two groups. While it was hypothesized that the well-trained participants would experience a stronger beneficial effect of the pre-event CHO intake compared to the recreationally trained, due to greater insulin sensitivity, CHO uptake, and oxidation of exogenous CHO (King et al., 1988; Burelle et al., 1999; Greiwe et al., 1999; Bowden and McMurray, 2000), this was not the case. The lack of difference in responses may be due to the relatively short exercise protocol, which likely did not result in low glycogen stores, or that the effect of greater oxidation of exogenous CHO was not sufficient to result in a measurable difference in TTE. Based on our findings it does not seem to be necessary to take different pre-test precautions based on the training status of the participant when the goal is to assess TTE or the associated performance-determining variables during a LT test and a GXT.

RER, GLU, and [La^−^]b were noticeably different between the two groups. RER and [La^−^]b were significantly higher in the first three exercise bouts in the recreationally trained group compared to the well-trained group, indicative of an earlier reliance on CHO oxidation and anaerobic metabolism (Mikulski et al., 2008; Ramos-Jiménez et al., 2008). However, despite lower mean RER in the well-trained group of the current study, there was a clear pattern of increasing GLU concentration with increasing exercise intensities, compared to a much flatter line in the recreationally trained group. This is likely explained by greater gluconeogenesis and hepatic glycogenolysis in the well-trained participants (Emhoff et al., 2013). Thus, it seems to be the case that the well-trained participants in the current study had through training obtained a better ability to cover the increasing metabolic requirements of higher intensities via a greater capability for mobilization of GLU as an energy source while the recreationally trained participants most likely had to rely on muscle glycogen to a greater extent. Based on the findings of the present study GLU mobilization appears to be trainable and the differences related to training status are substantial and should be taken into considerations whenever this measurement is used. However, this difference in GLU kinetics did not influence the effect of CHO on endurance capacity.

### Strengths and Limitations

The use of real-life meals in the current study increases the ecological validity of the study findings, whiles it removed the option of blinding participants to the intervention. This might have influenced TTE, due to participants' expectations related to the effect of CHO intake. Related to the differences in RER, GLU, and [La^−^]b between groups, the absolute energy expenditure was higher in the well-trained group and thus, the energy requirements differed which might explain the findings. The differences in GLU mobilization should be further investigated with the same relative and absolute intensities in both groups. The trained group in the current study did have a lower than recommended daily CHO intake (Thomas et al., 2016), this could have influenced the assumption that their glycogen stores were close to saturated. Lastly, in the current study, BM and BMI were significantly higher in the recreationally trained group compared to the well-trained group, which could have had an influence on metabolism and subsequently on the effect of the pre-event meal independent of the effect of training status (Caballero and Wurtman, 1991; Spiegelman et al., 1992).

## Conclusions

A pre-event meal with a high relative CHO content led to longer TTE and higher vLpeak compared to exercising in a fasted state and exercising after a pre-event meal with a low relative CHO content both in well-trained and recreationally trained participants. In contrast, pre-event CHO intake did not have any significant effect on the main physiological performance-determining variables; $\overset{∙}{\text{V}}{\text{O}}_{2\text{peak}}$, LT2, OBLA, or WE in either group. In conclusion, pre-event CHO intake should be standardized in future testing practice both in research and sports practices when TTE or vLpeak is the main outcome variable but seems of less importance when determining the main performance-determining factors.

## Data Availability Statement

The dataset generated for this study is available on request to the corresponding author.

## Ethics Statement

Ethical review and approval was not required for the study on human participants in accordance with the local legislation and institutional requirements. The patients/participants provided their written informed consent to participate in this study.

## Author Contributions

MA, DN, AET, and ØS designed the study and interpreted the results. MA collected the data and wrote the first draft. MA and DN performed the data analysis. All authors revised the manuscript, approved the submitted version, and agreed to be accountable for all the aspects of the work.

## Conflict of Interest

The authors declare that the research was conducted in the absence of any commercial or financial relationships that could be construed as a potential conflict of interest.
